# TRIM69: a marker of metastasis and potential sensitizer to 5-Fluorouracil and PD-1 blockers in colon adenocarcinoma

**DOI:** 10.1186/s12876-023-02927-9

**Published:** 2023-08-31

**Authors:** Xiao-Jv Chi, Yi-Bei Song, Deng-He Liu, Li-Qiang Wei, An-Ran Zhao, Xin An, Zi-Zhen Feng, Xiao-Hua Lan, Yu-Meng Lv, Hong-jun Li, Dong Lan, Hui-Min He

**Affiliations:** 1 Department of Clinical Laboratory, the First Affiliated Hospital of Guangxi Medical University, Key Laboratory of Clinical Laboratory Medicine of Guangxi Department of Education, Guangxi Zhuang Autonomous Region, 6 Shuangyong Road, Nanning, 530021 China; 2 Guangxi Medical University, Guangxi Zhuang Autonomous Region, 22 Shuangyong Road, Nanning, 530021 China; 3 Department of Medical Oncology, the First Affiliated Hospital of Guangxi Medical University, Guangxi Zhuang Autonomous Region, 6 Shuangyong Road, Nanning, 530021 China

**Keywords:** TRIM69, NOD-like signaling pathway, 5-Fluorouracil, PD-1 blockers, COAD

## Abstract

**Background:**

Several proteins in the tripartite-motif (TRIM) family are associated with the development of colorectal cancer (CRC), but research on the role of TRIM69 was lacking. The present study examined the correlation between TRIM69 expression and colon adenocarcinoma (COAD).

**Methods:**

mRNA sequencing data for COAD patients was extracted from The Cancer Genome Atlas to analyze correlations between TRIM69 expression and patients’ clinical features as well as survival. Potential associations with immune cells and chemosensitivity also were predicted using various algorithms in the TIMER, Limma, clusterProfiler, GeneMANIA, and Gene Set Cancer Analysis platforms. Subsequently, polymerase chain reaction analysis and immunohistochemical staining were used to detect TRIM69 expression in COAD tissue samples from real-world patients.

**Results:**

TRIM69 expression was lower in COAD tissues than in normal tissues and correlated with the pathologic stage and metastasis (M category). Additionally, TRIM69 was found to be involved in several immune-related pathways, notably the NOD-like signaling pathway. These results suggest that high TRIM69 expression has the potential to enhance tumor sensitivity to 5-fluorouracil and programmed cell death protein 1 (PD-1) blockers.

**Conclusions:**

From our findings that TRIM69 expression was significantly reduced in COAD compared with non-cancer tissues and associated with pathologic stage and metastasis, we conclude that increasing TRIM69 expression and/or activity may help to improve therapeutic outcomes. Accordingly, TRIM69 represents a potentially valuable marker of metastasis and target for adjuvant therapy in COAD.

**Supplementary Information:**

The online version contains supplementary material available at 10.1186/s12876-023-02927-9.

## Background

Colorectal cancer (CRC) is the most common malignancy occurring in the gastrointestinal tract and causes approximately 600,000 cancer deaths annually worldwide [1]. CRC is categorized according to several types, and colon adenocarcinoma (COAD) is the most prevalent type. Known risk factors for CRC include aging, alcohol consumption, obesity, smoking, and high meat intake [2]. Over the past few decades, major advances in precision therapy for CRC have greatly increased the survival time of patients [3]. Currently, the primary treatment consists of surgery combined with adjuvant chemotherapy and/or targeted drug therapy. Because most cases of CRC are diagnosed at an advanced stage and frequently with distant metastases, long-term survival remains unsatisfactory for the majority of patients [4]. For the development of individualized treatment plans and review schedules for CRC patients, clinicians need accurate information about patients’ condition. Therefore, research to identify new molecular biological markers and therapeutic targets for CRC is still needed.

The tripartite-motif (TRIM) protein family, also called as the RING, B-Box and coiled-coil (RBCC) protein family, consists of proteins containing tripartite motifs. Most TRIM proteins function as E3 ubiquitin ligases to degrade target proteins [5]. In recent studies, proteins of the TRIM family have been found to play crucial roles in a wide variety of pathological conditions, including inflammation, infectious disease, and cancer, and their interaction with p53 also contributes to these conditions [6]. In CRC, several TRIM family proteins have also been reported to be related to tumor cell proliferation, apoptosis, and metastasis [7–9]. The novel gene *TRIM69* was cloned from a cDNA library derived from human testes. Like other TRIM protein family members, TRIM69 contains the classical RBCC structural domain [10]. Although TRIM69 has been identified in various processes and pathways related to tumorigenesis, to our knowledge, the involvement of TRIM69 in tumor development remains incompletely understood and has not been studied in CRC.

For the present study, we downloaded transcriptomic data from The Cancer Genome Atlas (TCGA) for COAD for analysis of TRIM69 expression in conjunction with patients’ clinical characteristics. Subsequently, tumor and paraneoplastic tissues from COAD patients collected during clinical consultation were subjected to real-time quantitative reverse transcription PCR (RT-qPCR) analysis and immunohistochemistry to detect TRIM69 expression at different levels. Our analyses determined that TRIM69 expression was significantly lower in COAD tissue than in normal tissues and correlated with pathologic stage and metastasis (M category). Therefore, TRIM69 may serve as a marker of COAD and provide an adjuvant mechanism to enhance the effectiveness of various anti-cancer chemotherapeutic agents, such as 5-fluorouracil and programmed cell death protein 1 (PD-1) inhibitors.

## Methods

### Differential expression analysis

The mRNA expression data downloaded from TCGA-COAD included 524 mRNA expression profiles in total, including 482 for COAD tissues and 42 for normal colon tissues. To identify correlations between TRIM69 expression and clinical characteristics of COAD, Limma (version 3.52.4) was used to calculate correlation coefficients for TRIM69 expression and age, pathologic stage, and pathologic TNM stages. We also calculated the Pearson’s correlation coefficient for TRIM69 expression and TMB. Additionally, the TRIM69 expression matrix was extracted for differential expression analysis as well as survival analysis.

### Immune cell infiltration prediction

In TIMER 2.0, 10,897 samples representing 32 types of cancer were analyzed to determine whether different immune cell types have infiltrated the tumors. TIMER, quanTIseq, CIBERSORT, xCell, TIDE, EPIC and MCP-counter algorithms were used to calculate the predicted immune cell content [11]. Spearman analysis was applied to estimate the purity of tumor cells, and the “gene” module was further used to investigate the relationship between TRIM69 expression and infiltration of immune cells, including CD8 + T cells, CD4 + T cells, B cells, dendritic cells, macrophages, and neutrophils.

### GSEA

According to the mean expression of TRIM69 in patients, COAD patients were divided into high-expression and low-expression group. The Limma package was used to identify the genes differentially expressed in the high- and low-expression TRIM69 groups [12]. The clusterProfiler version 3.16.0 package was used for GSEA [13].

### Protein–protein interaction network

GeneMANIA is an online platform for analyzing gene lists, generating hypotheses regarding gene function, and prioritizing genes for functional studies [14]. In this study, proteins that interact with TRIM69 were identified using GeneMANIA.

### Over-representation analysis

TRIM69 and the genes found to interact with TRIM69 were input into g:Profiler [15]. Functional enrichment analysis was performed by g: GOSt. The following data sources were selected: GO Molecular Function, GO Cellular Component, GO Bioprocess, KEGG [16–18], Reactome and WikiPathways. GO and KEGG (DAVID tools, https://david.ncifcrf.gov/) enrichment analyses were performed for the top genes.

### Co-expression analysis

To identify genes that are highly correlated with TRIM69 and may be functionally related in tumor tissues, partial correlation coefficients were calculated. A partial correlation coefficient > 0.6 and *p* < 0.001 were used as the filter conditions. The results were visualized with the ggplot2 version 3.3.6 package.

### Chemotherapeutic drug sensitivity analysis

Within the Gene Set Cancer Analysis (GSCA), an integrated platform for the analysis of genomic, pharmacogenomic, and immunogenomic gene sets in cancer [19], the Drug module was used to calculate drug sensitivity among tumor cells according to the level of TRIM69 expression based on data from the Genomics of Drug Sensitivity in Cancer (GDSC) and The Cancer Therapeutics Response Portal (CTRP) projects.

### IPS for CTLA‑4 and PD‑1 blockers

Immune checkpoint blocking is an important strategy in anti-tumor immunotherapy. The Cancer Immunome Database (TCIA) provides systematic immunogenomic analyses of 20 solid tumors using data from TCGA. In this study, the IPS for CTLA-4 and PD-1 blockers for 457 COAD patients were downloaded from TCIA for prediction of the sensitivity to immune therapy.

### Acquisition of tissue samples and RT-qPCR analysis

TRIM69 mRNA expression was detected by RT-qPCR analysis in cancerous and paraneoplastic tissues from 141 COAD real-world patients. All tissue specimens were obtained from patients treated in the Department of Medical Oncology of the First Affiliated Hospital of Guangxi Medical University between February 2022 and June 2022. This project was approved by the Ethical Approval Committee of the First Affiliated Hospital of Guangxi Medical University (No. 2022-KT-transverse item-029), and the privacy of patients was well protected throughout this project.

We extracted the total RNA from tissues using RNAiso Plus reagent (Takara, Dalian, China), and mRNA was reverse transcribed into cDNA using the PrimeScript TM RT kit (Takara). Then, RT-qPCR was performed using SYBR® Premix Ex TaqTM II (Takara). The primer sequences in this analysis are presented in Table 1, and β-actin was used as the endogenous reference for normalization. Relative expression was calculated using the 2 ^– ΔΔCt^ method [20]. In addition, each experiment was performed three times.

**Table 1:** Sequences of primers used for RT-qPCR

Gene	Forward primer	Reverse primer
β-actin	AGTTGCGTTACACCCTTTCTTG	GCTGTCACCTTCACCGTTCC
TRIM69	GCATTCAGGCAAAGACGGAACAAC	ACTTCCCAGTACCACTTTCCAGAGG

### Immunohistochemistry

TRIM69 expression was detected by immunohistochemistry in cancerous and paracancerous normal tissues from 4 patients with COAD. All tissue samples were obtained from patients treated in the Department of Medical Oncology of the First Affiliated Hospital of Guangxi Medical University between February 2022 and June 2022. After deparaffinizing the slides for 20 min at 80 °C, they were immersed three times in a xylene bath for 5 min each time. The slides were sequentially washed twice with 100% ethanol for 30 s, 95% ethanol for 30 s and 75% ethanol for 30 s. Then they were placed in a pressure cooker set to high pressure for 5 min. After rinsing with phosphate-buffered saline (PBS), the slides were incubated overnight at 4 °C with the primary antibody (ABclonal) diluted in PBS. Following the overnight incubation at 4 °C, the slides were rinsed in PBS and incubated with secondary antibodies for 30 min at 37℃.

To conduct semi-quantitative analysis of immunohistochemistry, the following criteria were used. Based on staining intensity, the cells were scored as follows: 0 negatively stained, 1 weakly stained (pale yellow), 2 moderately stained (brownish yellow), and 3 strongly stained (sepia). Based on the percentage of positive cells, the positive ratio scores are as follows: 1, 25% or less; 2, 26–50%; 3, 51–75%; 4, 76% or more. Finally, we calculated the immunohistochemical score by multiplying staining intensity scores and positive ratio scores.

### Statistical analysis

A two-sided test of statistical significance was performed using the mean and standard deviation of the measurements as well as *p* < 0.05 for statistical significance. We compared the differences between two groups and multiple groups using Student’s t-test and one-way analysis of variance (ANOVA), respectively. The Chi-square test or Fisher’s exact probability method was used for comparisons of categorical data. R 4.2.1 and GraphPad (version 8.0) were used for statistical graphing.

## Results

### TRIM69 expression is reduced in COAD and associated with several clinical characteristics of COAD patients

**Fig. 1:**
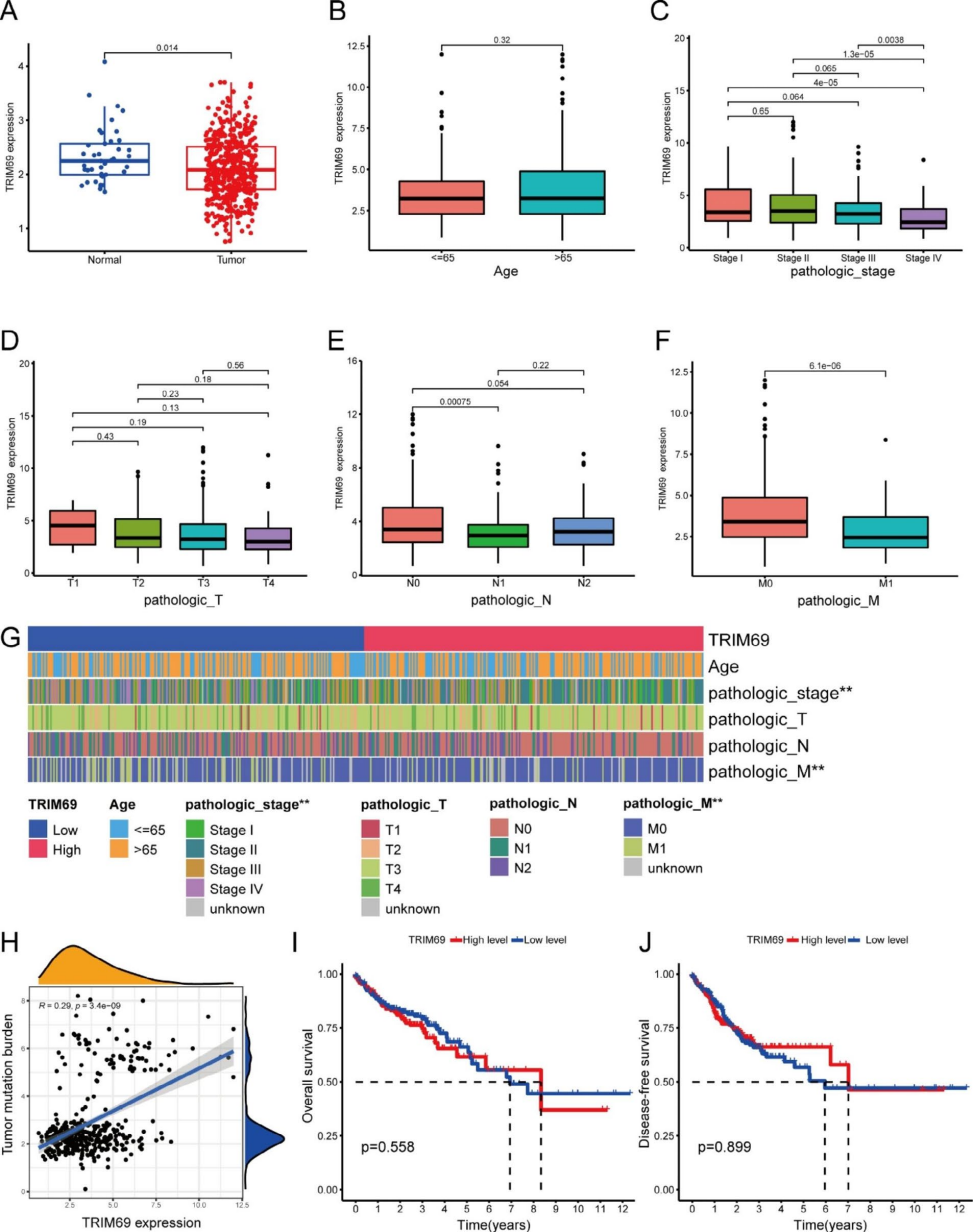
TRIM69 expression in patients with COAD within TCGA. (**A**) TRIM69 expression is lower in COAD tissue than in normal tissue. (**B-F**) Box plots and (**G**) heatmap for the associations between TRIM69 expression and the clinical characteristics of COAD cases. (**H**) Correlation of TMB and TRIM69 expression. (**I**) Overall survival among patients with high or low TRIM69 expression in COAD tissues. (**J**) Disease-free survival among patients with high or low TRIM69 expression in COAD tissues. (*p < 0.05; **p < 0.01; and ***p < 0.001)

Based on data from TCGA, comparison of TRIM69 expression in COAD tissues and non-cancer tissues revealed that the level of TRIM69 expression in COAD was significantly lower than that in non-cancer tissues (*p* < 0.05; Fig. 1A). Significant differences in TRIM69 expression also were observed across pathologic stages and M categories (*p* < 0.05; Fig. 1C, F **and G**). TRIM69 mRNA expression decreased with increasing pathologic stage, with the lowest level of TRIM69 mRNA expression observed in pathologic stage IV cases. Indeed, TRIM69 mRNA expression was significantly lower in stage IV COAD than in the other pathologic stages. Furthermore, as demonstrated by the results in Fig. 1H, TRIM69 expression in COAD was positively correlated with the tumor mutation burden (TMB).

TRIM69 expression did not vary significantly according to differences in age, pathologic T stage or pathologic N stage (Fig. 1B, D and E). Additionally, there was no statistically significant association between TRIM69 expression and overall survival (OS) or disease-free survival (DFS; Fig. 1I-J).

### TRIM69 expression is positively correlated with immune cell infiltration

**Fig. 2:**
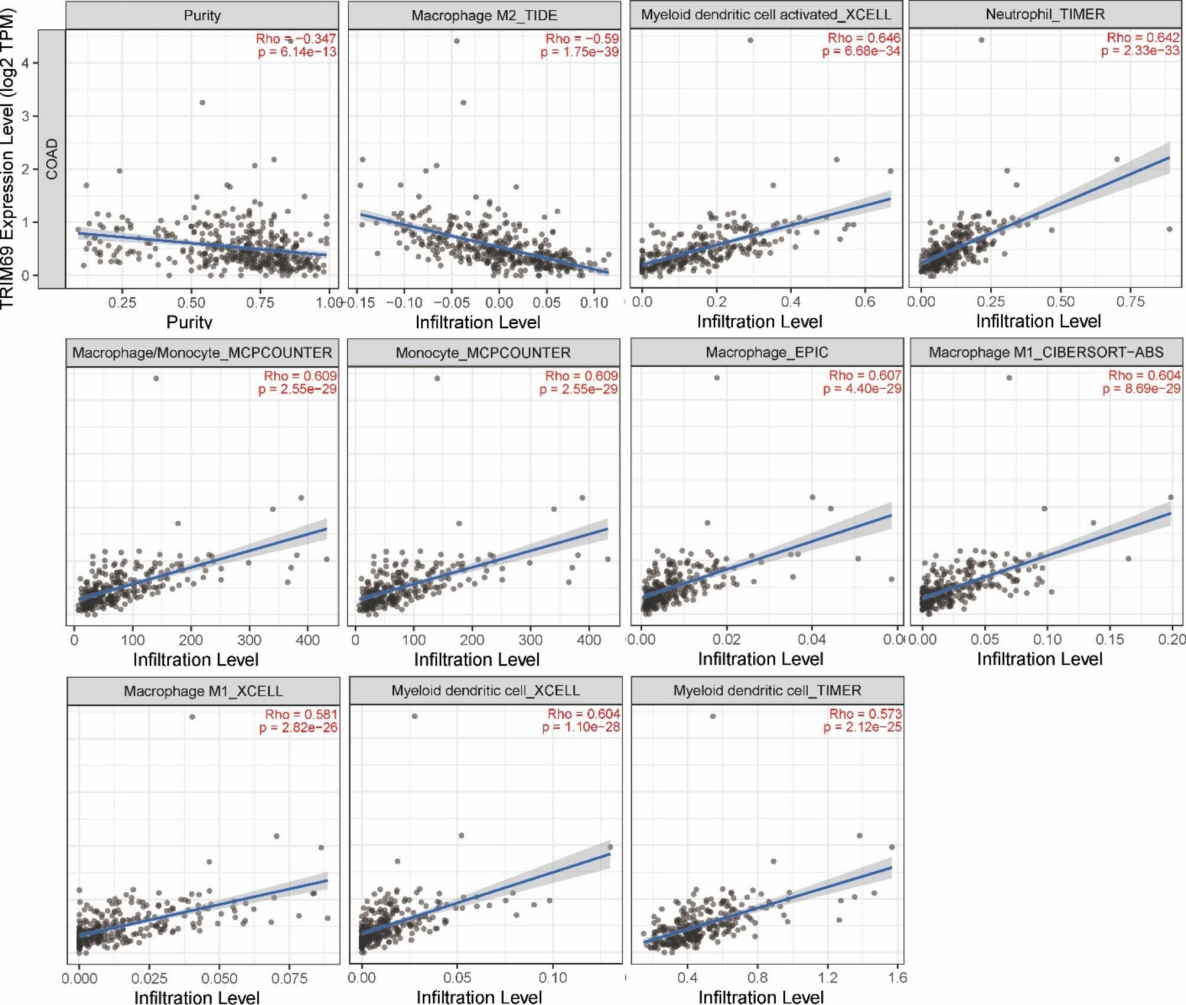
TRIM69 expression is positively correlated with the infiltration of multiple immune cell types in COAD

Previous studies have shown that several TRIM protein family members, including TRIM69, are involved in immune responses to viruses [21, 22]. To investigate the relationship between TRIM69 expression and immune cell infiltration in COAD, data from the TIMER database were used. As shown in Fig. 2, the top 10 most significant positive correlation were observed between TRIM69 expression and infiltration of COAD tissue by various immune cells, including activated myeloid dendritic cells, neutrophils, and monocytes (see Supplementary Table S1 for detailed results for immune cell infiltration prediction).

### TRIM69 expression is associated with altered gene expression and protein interaction

**Fig. 3:**
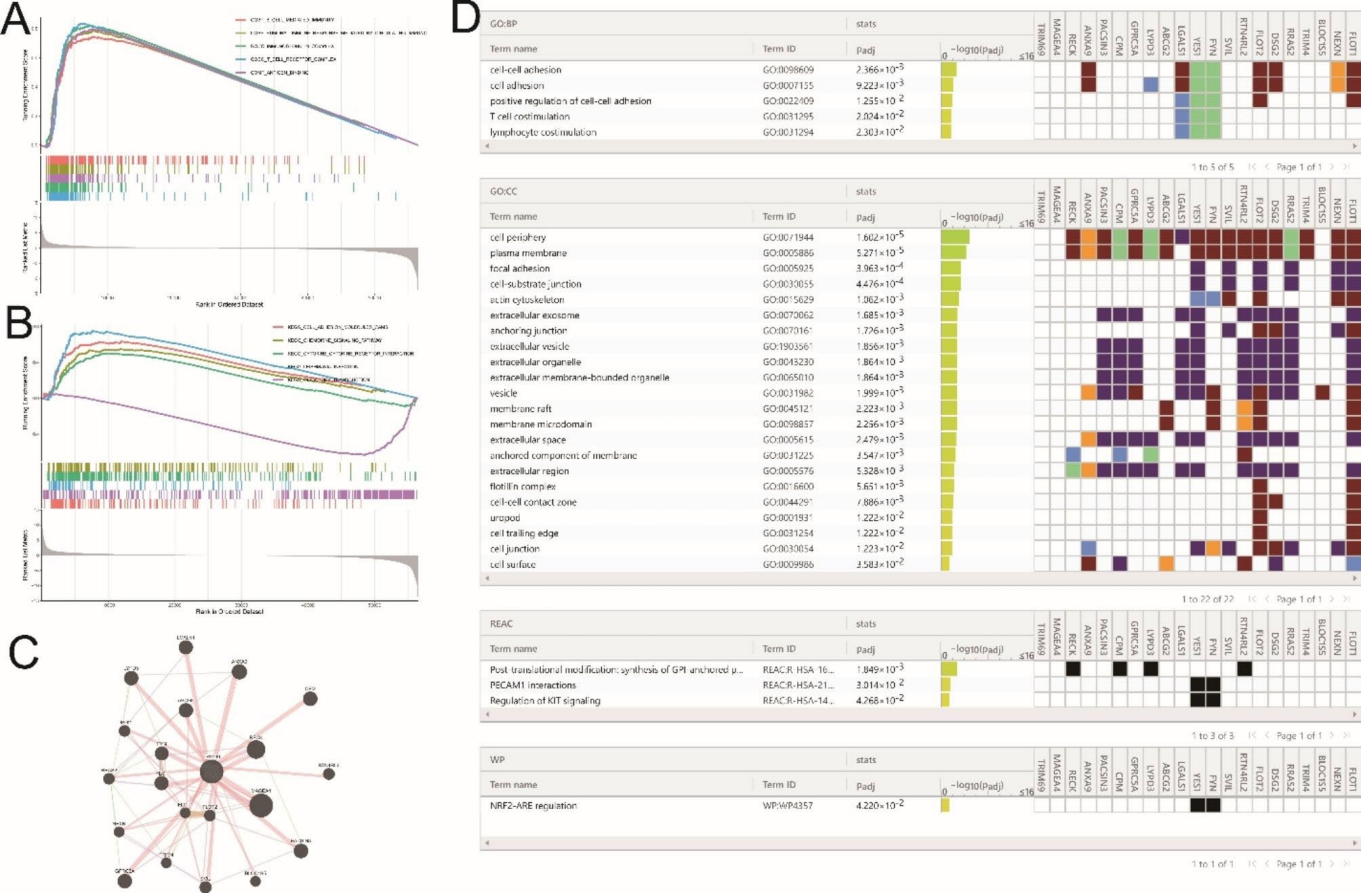
Enrichment analysis and protein interaction networks. (**A-B**) GSEA assessment of GO and KEGG terms associated with TRIM69 expression in COAD. (C-**D**) Protein interaction network for TRIM69 and overexpression enrichment analysis

Through gene set enrichment analysis (GSEA), some Gene Ontology (GO) and Kyoto Encyclopedia of Genes and Genomes (KEGG) entries were identified as significantly associated with TRIM69 expression in COAD, including immune-related functions and pathways such as B cell-mediated immunity, circulating immunoglobulin-mediated humoral immune responses, T-cell receptor complexes, cell adhesion molecules, and chemokine signaling pathways (Fig. 3A-B). GeneMANIA identified 20 proteins that interact with TRIM69 (Fig. 3C). G: Profiler analysis showed that these proteins are mostly associated with cell–cell adhesion, T cell co-stimulation, and lymphocyte co-stimulation (Fig. 3D). These results are consistent with our immune infiltration analysis showing an association between TRIM69 expression and the infiltration of multiple immune cell types.

### TRIM69 is linked to genes in the NOD-like receptor signaling pathway

**Fig. 4:**
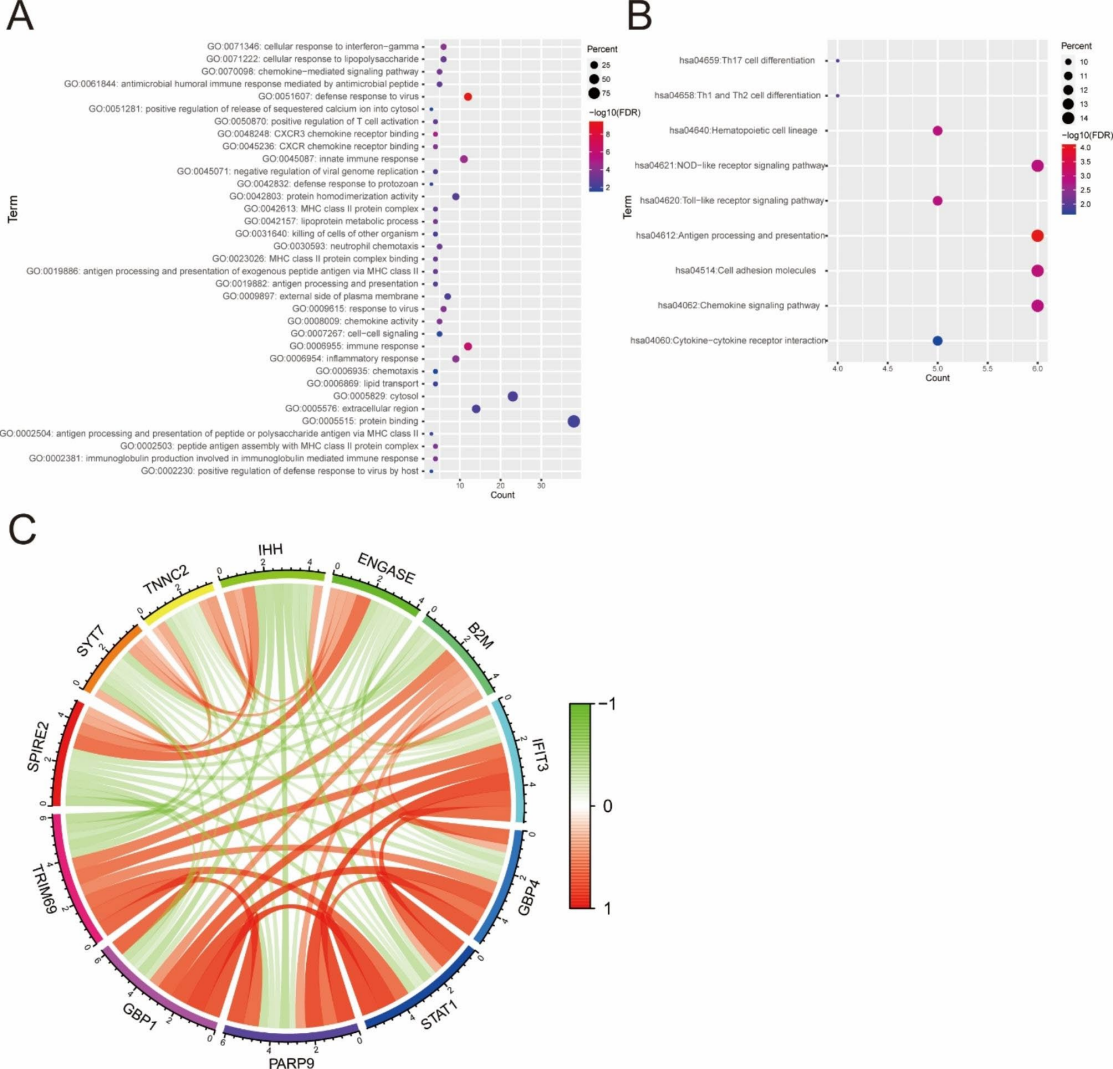
Co-expression analysis for TRIM69. (**A**) GO term enrichment analysis. (**B**) KEGG term enrichment analysis. (**C**) Circle plots for co-expression analysis of TRIM69 (positive correlation, red; negative correlation, green)

Gene co-expression analysis was carried out to construct a co-expression network. In total, 41 genes were identified that met the criteria **(**Table 2**)**. The results of GO and KEGG enrichment analyses are presented in Fig. 4A-B. The GO terms reflected genes that mainly contribute to protein binding, cytosol, and immune response. KEGG pathway enrichment analysis indicated that these genes are enriched in the NOD-like receptor signaling pathway, antigen processing and presentation, chemokine signaling pathway, and cell adhesion molecules. Together these findings indicate that the tumor microenvironment might be regulated by these genes in COAD.

**Table 2:** Genes for which expression correlated with TRIM69 expression in COAD

	Gene	Cor.	*p*
TRIM69	LAP3	0.645529357	6.27E-58
TRIM69	CXCL9	0.647065289	2.77E-58
TRIM69	B2M	0.69695384	4.89E-71
TRIM69	RARRES3	0.666031576	7.68E-63
TRIM69	OAS2	0.651602309	2.41E-59
TRIM69	CD8A	0.637904172	3.39E-56
TRIM69	USP18	0.621933898	1.01E-52
TRIM69	HLA-DMA	0.625152399	2.09E-53
TRIM69	CIITA	0.605650167	2.21E-49
TRIM69	IRF1	0.659994695	2.35E-61
TRIM69	DDX60	0.646022106	4.83E-58
TRIM69	APOL1	0.609288223	4.12E-50
TRIM69	NKG7	0.607489827	9.48E-50
TRIM69	HLA-DRA	0.630983128	1.15E-54
TRIM69	PARP9	0.745890995	2.14E-86
TRIM69	GBP4	0.714694398	3.14E-76
TRIM69	SAMD9L	0.667592348	3.13E-63
TRIM69	IFIT5	0.608436777	6.12E-50
TRIM69	APOL6	0.656752869	1.43E-60
TRIM69	TRIM22	0.62420561	3.32E-53
TRIM69	CD2	0.615986329	1.76E-51
TRIM69	HLA-DMB	0.635238189	1.33E-55
TRIM69	CD274	0.622386901	8.08E-53
TRIM69	BTN3A3	0.621567972	1.20E-52
TRIM69	WARS	0.671522192	3.18E-64
TRIM69	CCL5	0.632111933	6.50E-55
TRIM69	CXCL11	0.660825362	1.48E-61
TRIM69	GZMA	0.664524822	1.82E-62
TRIM69	GBP2	0.629875921	2.00E-54
TRIM69	APOBEC3G	0.633545712	3.15E-55
TRIM69	IFIT2	0.616308356	1.51E-51
TRIM69	PARP14	0.611333312	1.59E-50
TRIM69	UBE2L6	0.654568941	4.77E-60
TRIM69	CXCL10	0.691406465	1.72E-69
TRIM69	APOL2	0.610983809	1.87E-50
TRIM69	IFIT3	0.713083505	9.65E-76
TRIM69	GBP1	0.755542382	7.55E-90
TRIM69	IDO1	0.681818394	6.73E-67
TRIM69	STAT1	0.719246902	1.26E-77
TRIM69	CXCL13	0.616064991	1.70E-51
TRIM69	APOL3	0.619396557	3.44E-52

The co-expression plot in Fig. 4C shows the relationship between TRIM69 expression and the top 5 negatively (green; *GBP1*, *PARP9*, *STAT1*, *GBP4*, and *IFIT3*) and top 6 positively (red; *B2M*, *SPIRE2*, *SYT7*, *TNNC2*, *IHH*, and *ENGASE*) correlated genes. These genes were enriched in the NOD-like receptor signaling pathway. The above results led to the hypothesis that TRIM69 might influence the tumor immune microenvironment by regulating NOD-like receptor signaling.

### TRIM69 expression is associated with tumor sensitivity to multiple drug compounds

**Fig. 5:**
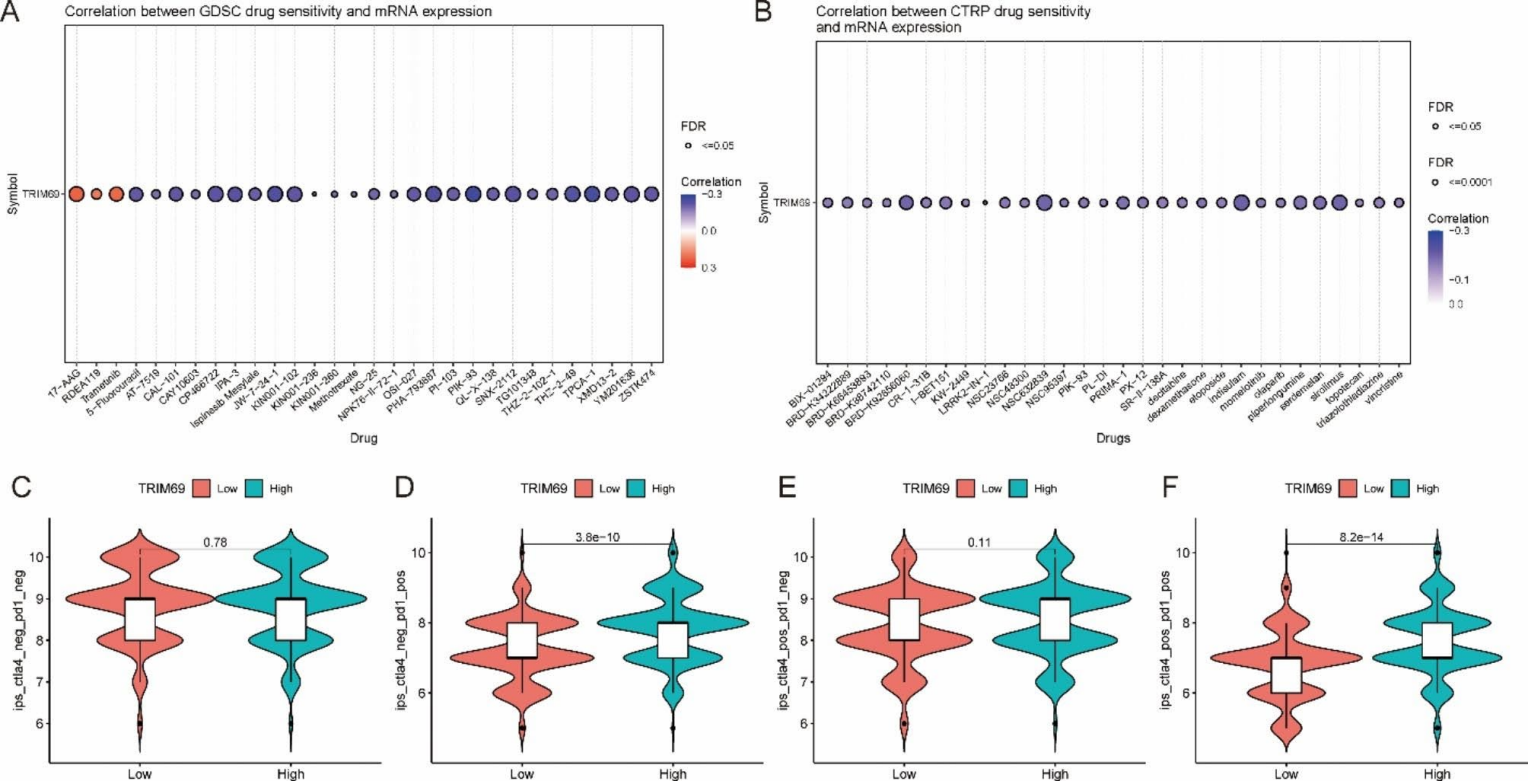
Relationships between TRIM69 expression and the efficacy of oncology chemotherapeutics (IC_50_). (**A**) Correlation between sensitivity to GDSC and TRIM69 mRNA expression. (**B**) Correlation between sensitivity to CTRP and TRIM69 mRNA expression. (**C-F**) IPS values for CTLA-4 and PD-1 blockers in 457 patients with COAD

Gene Set Cancer Analysis (GSCA) was applied to analyze the sensitivity of tumor cells to different anti-cancer drugs according to the level of TRIM69 expression. TRIM69 expression was significantly positively correlated with the half maximal inhibitory concentration (IC_50_) values for 17-AAG, RDEA119, and trametinib, as well as significantly negatively correlated with the IC_50_ values for 5-fluorouracil, AT-7519, CAL-101, and indisulam (Fig. 5A-B). These results suggest that increased TRIM69 expression could increase the sensitivity of colon cancer cells to 5-fluorouracil, AT-7519, and CAL-101.

### TRIM69 expression is associated with the response to immunotherapy

We compared the immunotherapy scores (IPSs) for CTLA-4 and PD-1 blockers between groups of COAD patients with high and low TRIM69 expression. As shown in Fig. 5C-F, patients with positive PD-1 status in the high-expression TRIM69 group had good responses to a PD-1 inhibitor.

### TRIM69 expression is lower in COAD tissues than in normal tissues

**Fig. 6:**
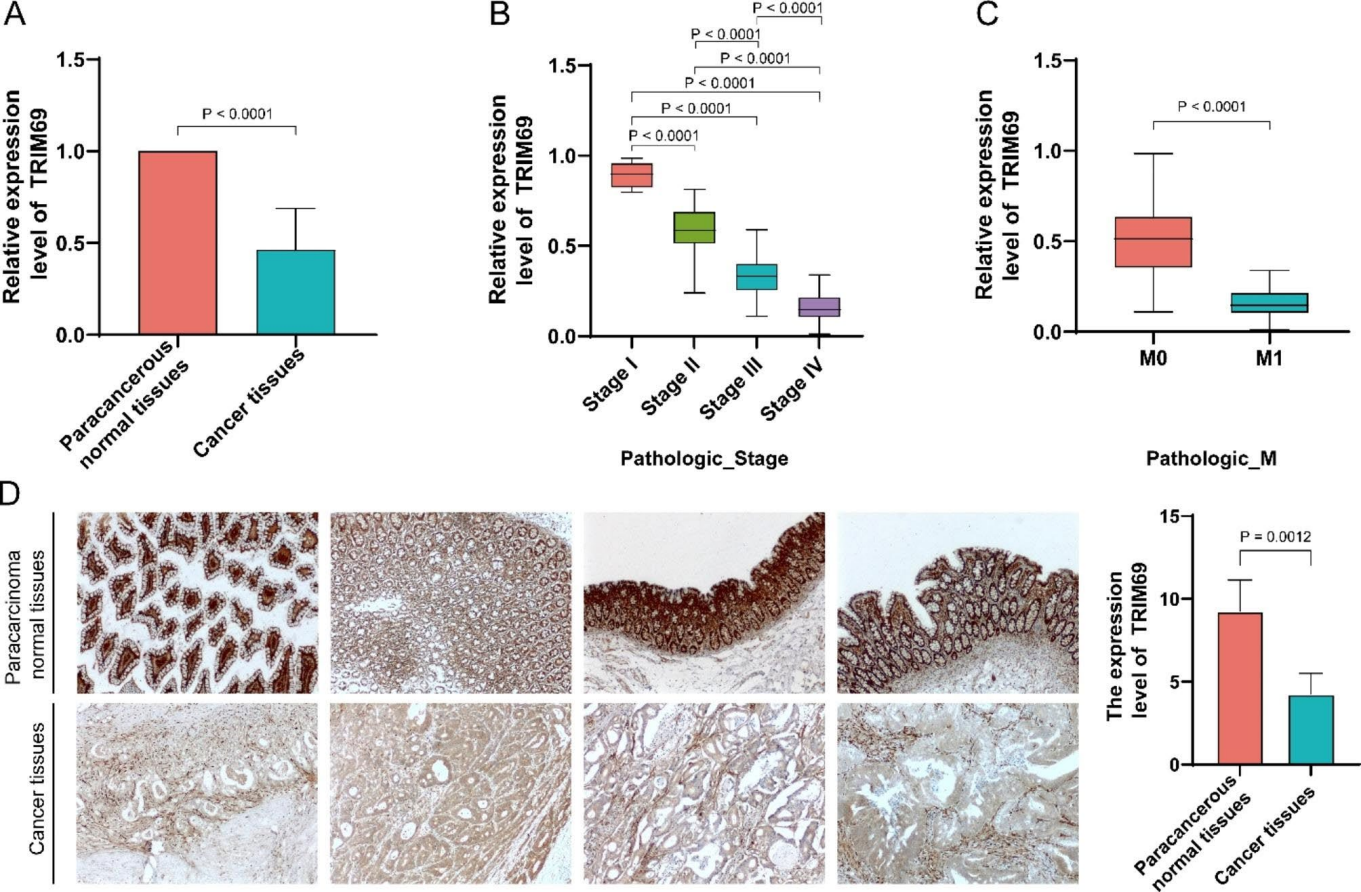
Expression of TRIM69 was verified at the mRNA and protein levels in the 141 patients with COAD. (**A**) Relative TRIM69 mRNA expression levels in COAD tissues and normal tissues by RT-qPCR. Relative TRIM69 mRNA expression levels in different pathologic stage (**B**) and different M stages (**C**) of real-world patients with COAD. (**D**) TRIM69 expression in COAD tissues and normal tissues observed by immunohistochemical staining

Based on RT-qPCR analysis of tumor tissues and paracancerous normal tissues from 141 COAD patients, TRIM69 expression was significantly lower in tumor tissues than in non-malignant paracancerous normal tissues (Fig. 6A) and was markedly different in the cancer tissues of different pathological stages and different M stages (Fig. 6B-C), consistent with results found from analysis of COAD patient data from TCGA database (see Supplementary Figure S1 for detailed results for age, different T stages and different N stages).

The results of immunohistochemical staining analysis and immunohistochemical scoring (Fig. 6D) supported the findings from RT-qPCR for TRIM69 different expression between tumor tissues and normal tissues.

## Discussion

A number of factors are believed to contribute to COAD development, including oncogenetic activation and deletion of cancer suppressor genes [23]. In recent years, several TRIM family proteins have been suggested to be upregulated or downregulated and to play important roles in COAD. For example, hypoxia-induced upregulation of TRIM14 was shown to inhibit the proliferation and invasion of HCT116, SW480, HT29, and LoVo cells [7]. Additionally, TRIM21 expression was found to be reduced in cancers associated with colitis and to negatively regulate intestinal epithelial carcinogenesis by modulating epithelial cell proliferation, adhesion, tissue remodeling, and angiogenesis as well as pro-inflammatory responses [24]. TRIM15, TRIM52, and TRIM67, among others, have been reported to be associated with the malignancy of COAD [8, 25, 26].

To our knowledge, the specific functional role of TRIM69 in the development of COAD had not been examined. A study of non-small cell lung cancer (NSCLC) demonstrated that TRIM69 is associated with spindle poles and promotes centrosome clustering, which is essential for the formation of bipolar spindles, and that mitotic arrest caused by silencing TRIM69 inhibits tumor growth in vivo [27]. Additional studies have shown that TRIM69 is involved in numerous processes and pathways underlying tumorigenesis. Rong et al. showed that upon silencing of TRIM69, apoptosis and ROS production are promoted, and TRIM69 interacts with p53 and induces its ubiquitination [28]. The results of the present study indicate that TRIM69 is differentially expressed in different tissue types and in tumor tissues according to pathologic stage or M stages, albeit not statistically associated with OS or DFS. Therefore, we speculate that TRIM69 may play an important role in the development of COAD.

Invasion and metastasis are not only the basic biological characteristics of tumors but are key factors that promote the progression of malignant tumors [29]. COAD patients were grouped according to clinical traits to investigate TRIM69 expression correlated with differences in these traits. From this analysis, lower expression of TRIM69 was detected in COAD tissues than in non-cancer tissues by RT-qPCR and immunohistochemistry. TRIM69 expression also correlated with the pathologic stage and M category of COAD, suggesting that TRIM69 may serve as a marker of metastasis in COAD and potentially be a new colon cancer suppressor. The results of our experiments did suggest that the level of TRIM69 expression may influence the response to different therapies for COAD.

Given the molecular etiology of COAD, appropriate selection of drug therapy is likely important for these patients. Although immune checkpoint drugs and chimeric antigen receptor T-cell therapies have greatly improved survival for some cancer types, progress related to the clinical application of immunotherapies for COAD has been slow [30]. 5-Fluorouracil remains the standard first-line chemotherapeutic agent used to treat COAD. However, in most cases, tumors that initially respond to 5-fluorouracil therapy eventually develop chemoresistance [31]. In this study, we found that TRIM69 expression was negatively correlated with the IC_50_ of several chemotherapy drugs, including 5-fluorouracil, AT-7519, CAL-101, and indisulam. This suggests that increasing TRIM69 expression might enhance the therapeutic effects of various anticancer drugs. We also found that TRIM69 expression influenced the IPS of PD‑1 blockers in COAD. Thus, PD-1 positive patients with high-level TRIM69 expression are more likely to benefit from PD‑1 blockers. In this way, our findings provide insight for a potentially novel therapeutic strategy for COAD.

As a potent inhibitor of vesicular stomatitis virus (VSV) infection, TRIM69 regulates the innate immune suppression of a range of viral infections [32]. TRIM69 restricts the replication of dengue virus by specifically ubiquitinating the non-structural proteins of the virus [33]. We initially explored the possible role of TRIM69 in tumor immunity by using the TIMER2.0 database and GSEA and observed a significant positive correlation between TRIM69 expression and the infiltration of several immune cell types, including activated myeloid dendritic cells, neutrophils, and monocytes. The GSEA results also revealed that TRIM69 may be involved in numerous immune-related processes and pathways in COAD. Still, whether TRIM69 can inhibit tumor development through immune-related effects remains to be confirmed by further studies.

To study the specific signaling pathways underlying the role of TRIM69 in COAD, we first performed co-expression analysis to define key regulatory genes. The results of GO and KEGG enrichment analyses of key regulatory genes revealed their involvement in immune responses and related-signaling pathways. Additionally, KEGG analysis of the top genes showed their enrichment in the NOD-like receptor signaling pathway (*p* < 0.05). The NOD-like receptor signaling pathway plays a key role in pathogen recognition and the innate immune response. Although exact evidence for the relationship between TRIM69 and the NOD-like receptor-signaling pathway is lacking, we can speculate that TRIM69 may be involved in the anti-tumor response via the NOD-like receptor-signaling pathway.

## Conclusions

In conclusion, the present study showed that TRIM69 was significantly downregulated in COAD compared to non-cancer tissues, and the expression level of TRIM69 was associated with pathologic stage and metastasis. Consistent with this, the mRNA expression level of TRIM69 was low in tumor tissue. Notably, TRIM69 expression showed significant negative correlations with the IC_50_ values of various anti-cancer drugs, including 5-fluorouracil, AT-7519, CAL-101, and indisulam. Therefore, increasing TRIM69 expression or activity may help to improve therapeutic outcomes. Based on IPS calculations, PD-1 blockers may be an effective therapeutic strategy for COAD with higher TRIM69 expression. Our experiments also provide evidence that TRIM69 may improve the tumor immune microenvironment through the immune response, specifically the NOD-like receptor signaling pathway. However, further research is required to confirm the underlying mechanisms of TRIM69 function in COAD.

### Electronic supplementary material

Below is the link to the electronic supplementary material.

Supplementary Material 1

Supplementary Material 2

## Data Availability

The datasets analysed during the current study are available in the Genomic Data Commons Data Portal, [https://portal.gdc.cancer.gov/repository].

## Abbreviations

ANOVA: Analysis of variance
CRC: Colorectal cancer
COAD: Colon adenocarcinoma
DFS: Disease-free survival
GSCA: Gene Set Cancer Analysis
GO: Gene Ontology
GSEA: Gene set enrichment analysis
GDSC: Genomics of Drug Sensitivity in Cancer
CTRP: The Cancer Therapeutics Response Portal
GSCA: Gene Set Cancer Analysis
IPSs: Immunotherapy scores
KEGG: Kyoto Encyclopedia of Genes and Genomes
NSCLC: Non-small cell lung cancer
RBCC: RING, B-Box and coiled-coil
OS: Overall survival
TCGA: The Cancer Genome Atlas
TMB: Tumor mutation burden
TRIM: Tripartite-motif
VSV: Vesicular stomatitis virus
