# Supplementary material for: TRIM69: a marker of metastasis and potential sensitizer to 5-Fluorouracil and PD-1 blockers in colon adenocarcinoma
Source: BMC Gastroenterol. 2023 Aug 31;23:292. doi: 10.1186/s12876-023-02927-9 (PMC10470154; doi:10.1186/s12876-023-02927-9)

**Figure S1.** Expression of TRIM69 was verified at the mRNA level in the 141 patients with COAD. TRIM69 mRNA expression levels in different age groups (A) and cases with different T stages (B) and N stages (C) among real-world patients with COAD.


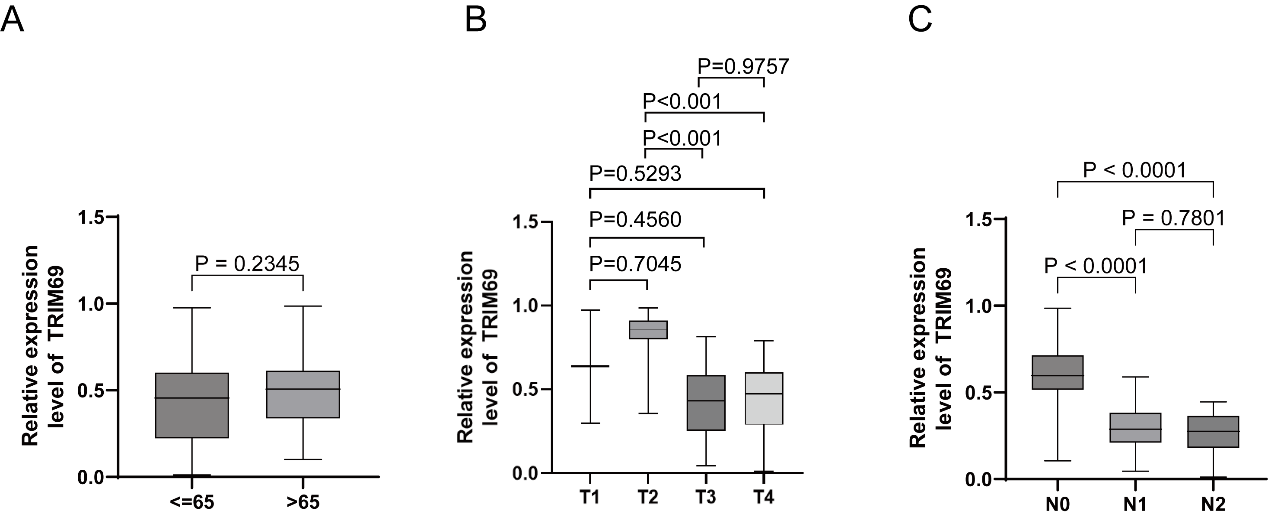

Supplement: Supplementary file 2 — Supplementary Material 2 [file 12876_2023_2927_MOESM2_ESM.docx]
